# Delta-9-tetrahydrocannabinol inhibits invasion of HTR8/SVneo human extravillous trophoblast cells and negatively impacts mitochondrial function

**DOI:** 10.1038/s41598-021-83563-9

**Published:** 2021-02-17

**Authors:** O’Llenecia S. Walker, Harmeet Gurm, Reeti Sharma, Navkiran Verma, Linda L. May, Sandeep Raha

**Affiliations:** 1 Graduate Program in Medical Sciences, Department of Pediatrics, McMaster University, HSC 4H7, Hamilton, ON L8S 4K1 Canada; 2 Department of Pediatrics, McMaster University, HSC 4H7, Hamilton, ON L8S 4K1 Canada

**Keywords:** Reproductive biology, Mitochondria

## Abstract

Prenatal cannabis use is a significant problem and poses important health risks for the developing fetus. The molecular mechanisms underlying these changes are not fully elucidated but are thought to be attributed to delta-9-tetrahydrocannabinol (THC), the main bioactive constituent of cannabis. It has been reported that THC may target the mitochondria in several tissue types, including placental tissue and trophoblast cell lines, and alter their function. In the present study, in response to 48-h THC treatment of the human extravillous trophoblast cell line HTR8/SVneo, we demonstrate that cell proliferation and invasion are significantly reduced. We further demonstrate THC-treatment elevated levels of cellular reactive oxygen species and markers of lipid damage. This was accompanied by evidence of increased mitochondrial fission. We also observed increased expression of cellular stress markers, *HSP70* and *HSP60*, following exposure to THC. These effects were coincident with reduced mitochondrial respiratory function and a decrease in mitochondrial membrane potential. Taken together, our results suggest that THC can induce mitochondrial dysfunction and reduce trophoblast invasion; outcomes that have been previously linked to poor placentation. We also demonstrate that these changes in HTR8/SVneo biology may be variably mediated by cannabinoid receptors CB1 and CB2.

## Introduction

The active components of *Cannabis sativa* have been used for centuries for both recreational and medicinal purposes^1,2^, and the clinical use of cannabis-based medicines is increasing worldwide^3^. Delta-9-tetrahydrocannabinol (THC) is the primary constituent of cannabis with pharmacological and toxicological effects^2^ mediated by the canonical G-protein coupled cannabinoid receptors CB1 and CB2 found in the central nervous system (CB1) and various peripheral tissues (CB1 and CB2)^4^, including the placenta^5,6^. Cannabis is commonly used amongst pregnant women^7–9^, purportedly due to the view that it is a natural product with therapeutic benefits, particularly its antiemetic and analgesic effects^10,11^. A small study surveyed pregnant women in Vancouver, Canada and found that 77% of respondents used cannabis to manage nausea^12^. This study demonstrates the propensity of women in certain demographics to use cannabis, but larger studies assessing cannabis use during pregnancy suggest that the numbers range from 2 to 20%^13,14^. Poor pregnancy outcomes are associated with maternal cannabis/THC use. Maternal circulating THC crosses the placenta to impact placental and fetal development^8^. While there are presently no reported teratogenic effects, cannabis has been heavily implicated in neurodevelopmental disorders in the offspring^15–17^, intrauterine growth restriction^18,19^, and preterm birth^19–21^. Though the pathology and the mechanism(s) underpinning the adverse fetal outcomes associated with maternal cannabis use during pregnancy have yet to be fully elucidated, they are thought to stem from trophoblast abnormalities^22–26^.

Trophoblasts are the main cell type of the placenta. Normal placentation requires invasion of extravillous trophoblasts (EVTs) into the maternal decidua and approximately the inner third of the myometrium^27^. This invasive process is tightly controlled both spatially (restricted to the decidua, inner third of the myometrium and the spiral arteries) and, temporally (primarily in the first trimester) by several interactions between the invading trophoblasts, the decidua, and the maternal vasculature and immune system^27^ (for review, see^28^). To facilitate invasion, trophoblasts secrete proteases, such as matrix metalloproteinases (MMPs), whose in vivo activity is regulated by their tissue inhibitors of MMPs (TIMPs)^27^. The balance between MMPs and TIMPs is critical for embryonic implantation, and when disturbed, can lead to various pregnancy complications, like PE^28^.

Oxidative stress occurring locally at the materno-fetal interface can lead to impaired trophoblast function, preventing physiological remodelling of the uterine spiral arteries, ultimately leading to shallow placental implantation; characteristic of PE^29^. Furthermore, placental oxidative stress is also linked to mitochondrial dysfunction^30,31^. Adequate mitochondrial function is essential in pregnancy because it sustains the increased metabolic activity of the placenta throughout gestation^31^. Mitochondrial dysfunction represents a critical physiological factor for fetal programming in cases of placental insufficiency^31^. In mouse embryos, mitochondrial dysfunction affects subsequent placentation and fetal growth^32^. Additionally, maternal undernutrition in rats induces impaired placental mitochondrial function and results in fetal and placental growth restriction^33^. Recently, associations between mitochondrial dysfunction and THC exposure have been suggested^34^. THC treatment in human lung cancer cells (H460) was shown to reduce mitochondrial complex I and complex II-III activities, reduce mitochondrial membrane potential, and induce oxidative stress and apoptosis^34^.

THC is thought to alter mitochondrial function in brain, muscle^35–37^, thymus, spleen^38^, and placental tissue^19,22^. The high energetic demands of a tissue like the placenta make it susceptible to agents that might attenuate mitochondrial function. In the process of electron transport to generate ATP, mitochondria can be a major source of reactive oxygen species (ROS). These ROS species also serve as important signaling molecules and can trigger cellular dysfunction or cell death by damaging proteins, lipids, and mitochondrial DNA. Therefore it is important for the mitochondria to have mechanisms to ensure the maintenance of healthy mitochondria^39^. Quality control can occur by fission and fusion to allow segregation of damaged mitochondria, mitophagy to remove damaged mitochondria, and cell death if the damage is too severe^39^. Decreased inner mitochondrial membrane potential, which is approximately -120 mV^40^, is an important trigger for mitochondrial fission. Indeed, we have shown that targeted perturbation of mitochondria resulted in reduced syncytiotrophoblast function and increased ROS production^41^. Based on the role of mitochondria in energy transduction, it is not surprising that any perturbations in mitochondrial energy production, or propagation would result in the development of placental pathology or susceptibility to placental damage^30,31,39^.

We hypothesized that exposure to 20 µM THC, within the range of concentrations measured in the serum of cannabis users^25,42^, for 48 h would lead to an inhibitory effect on the invasive capability of trophoblast cells. Using a first-trimester immortalized trophoblast cell line, HTR8/SVneo, a well accepted model of extravillous trophoblasts^43–45^, our study objective was to evaluate the effect of THC exposure on cell viability, MMP and TIMP expression, transwell invasion, alongside the assessment of mitochondrial function.

## Results

### 20 µM THC inhibits proliferative activity of HTR8/SVneo cells without damage to the plasma membrane

Figure S1 demonstrates that HTR8/SVneo proliferation, over 48 h, was reduced following exposure to THC concentrations ≥ 15 µM (MTS assay, *P* < 0.05), with a 50% reduction evident at 20 µM (*P* < 0.0001). Plasma membrane damage became evident only at 30 µM THC (increased LDH release into media, *P* < 0.05) when compared to unstimulated cells. We utilized 20 µM THC for treatments in subsequent studies.

### THC markedly reduced the invasive capacity of trophoblasts

**Figure 1:**
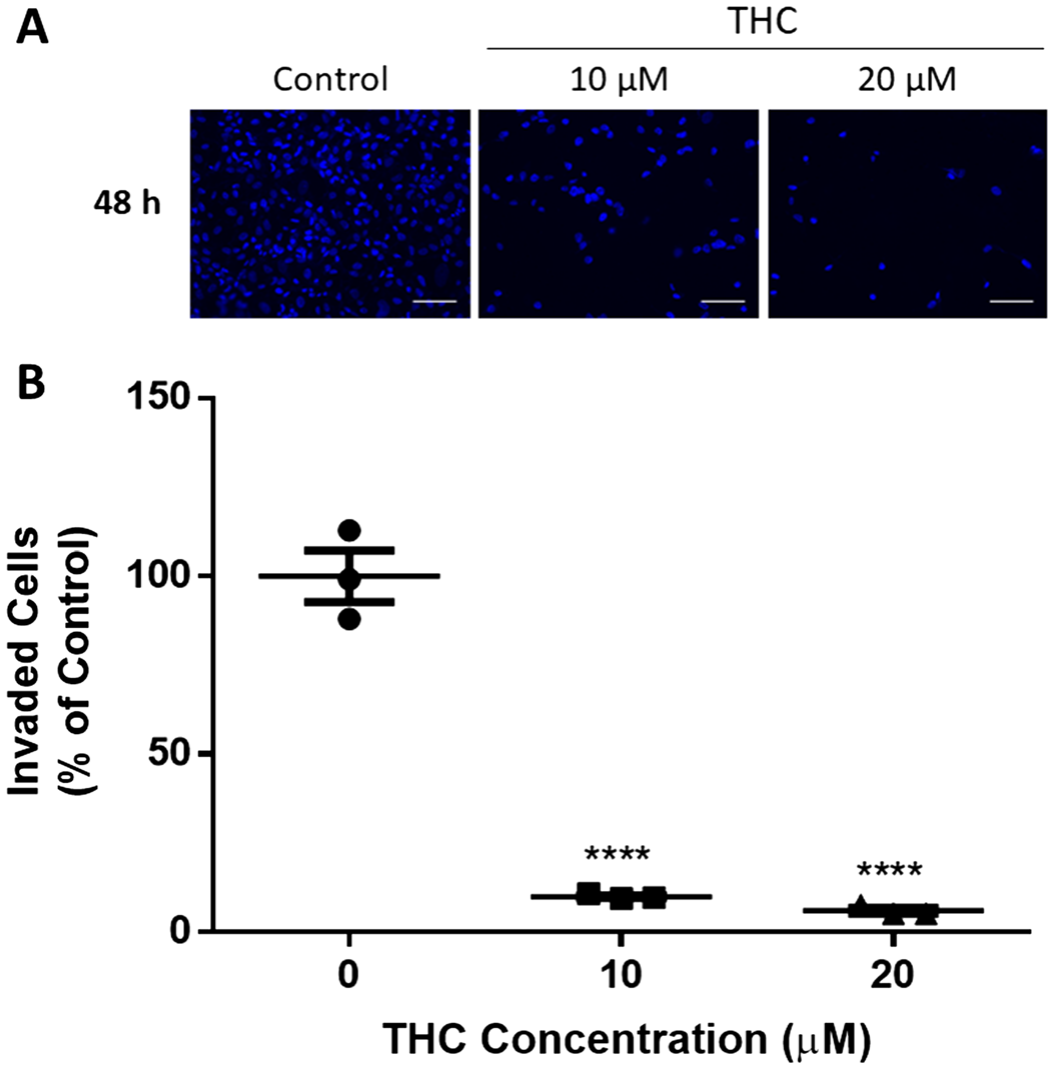
THC reduces HTR8/SVneo cell invasion. HTR8/SVneo cells were added to Matrigel-coated insert wells. The inserts were incubated for 48 h as described in “Methods”. **(A)** Representative immunofluorescent images of invaded HTR8/SVneo cells are shown, with the nuclei staining blue, magnification  × 200 and scale bar indicates 100 µm (Nikon Eclipse Ti-E). **(B)** A summary histogram reporting the percentage of invaded cells relative to the vehicle control group (methanol), each data point is shown, the horizontal lines represent the means and the error bars represent SEM (n = 3). Significant differences were determined by a one-way ANOVA, followed by a Bonferroni post hoc test. **** *P* < 0.0001 **(B)**.

The effect of THC on HTR8/SVneo invasion was determined using Matrigel-coated transwell inserts. The number of invasive HTR8/SVneo cells was profoundly reduced upon exposure to THC, as shown in Fig. 1 (*P* < 0.0001). We evaluated HTR8/SVneo invasion at 10 µM and 20 µM THC treatment. These two concentrations are well within physiological levels but had quite different effects on cell proliferation (Fig. S1A). We observed almost a 90% reduction in cell invasion using both concentrations of THC (*P* < 0.0001).

### THC exposure negatively alters MMP and TIMP transcript and protein expression

**Figure 2:**
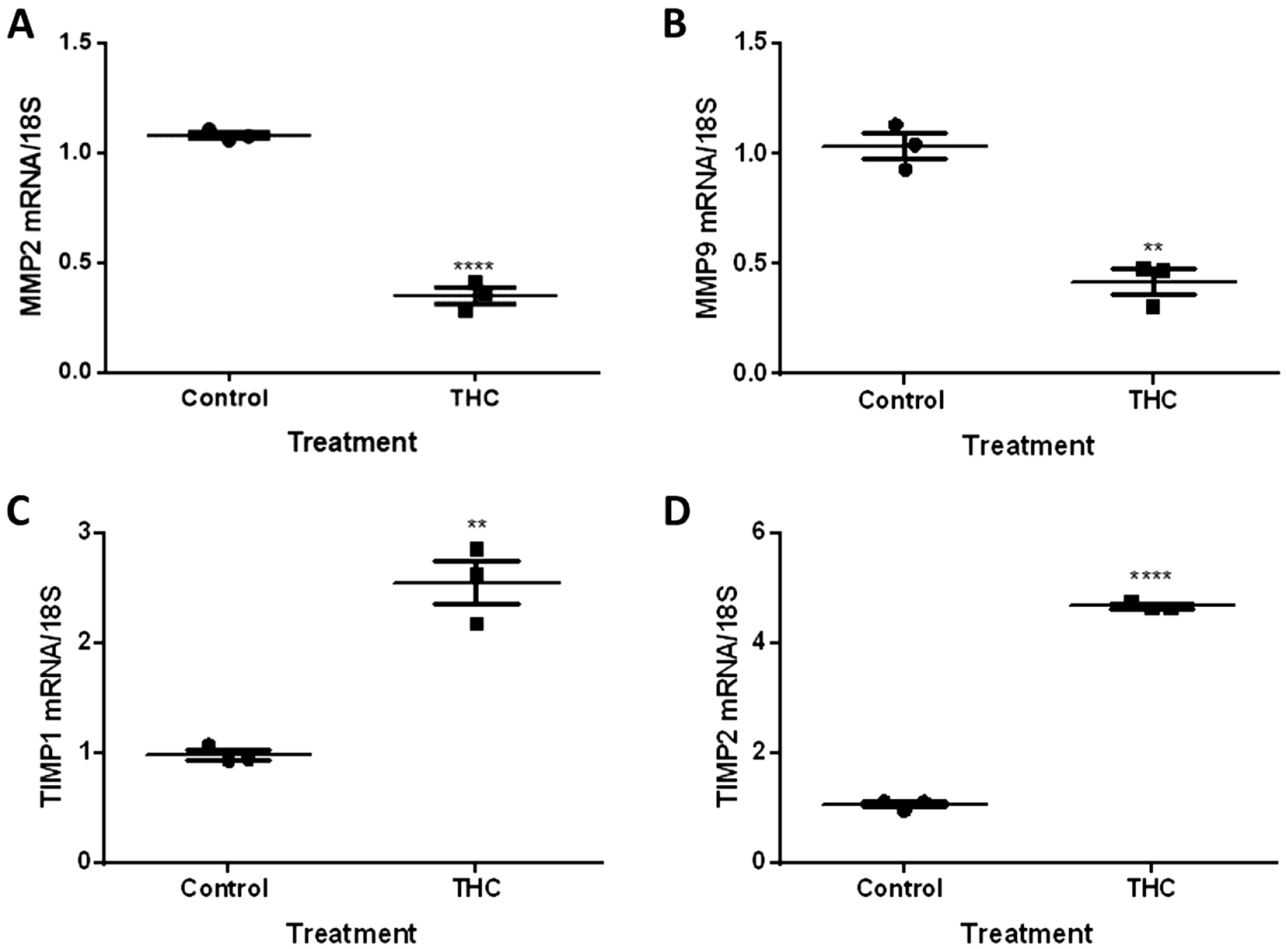
THC alters the mRNA expression of degradative enzymes and their inhibitors. HTR8/SVneo cells were treated for 48 h with 20 µM THC as described in the methods. Summary histograms of relative *MMP2* **(A)**, *MMP9* **(B)**, *TIMP1* **(C)**, and *TIMP2* **(D)** transcript expression in each treatment group normalized to 18S, then compared to the gene in the vehicle control. Each data point represents the mean ± SEM of 3 biological replicates. Significant differences were determined by Student’s t-test. **** *P* < 0.0001 **(A,D)**; ** *P* < 0.01 **(B,C)**.

**Figure 3:**
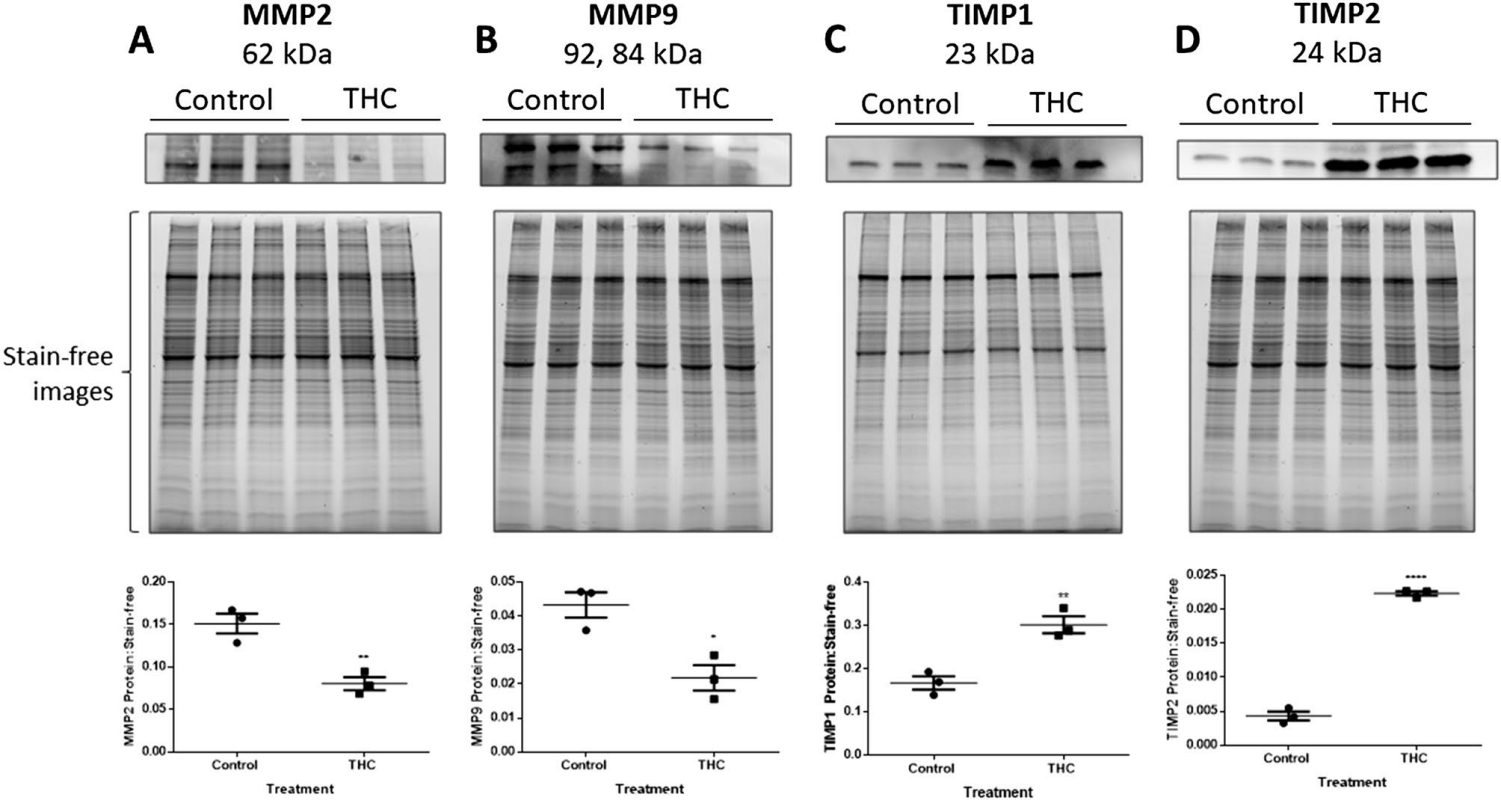
THC alters the expression of MMP and TIMP proteins in HTR8/SVneo cells. **(A–D)** Total protein was isolated from HTR8/SVneo cells and analyzed by Western blot (20 µg). Summary histograms of active MMP2 **(A)**, pro-MMP9 and active MMP9 **(B)**, TIMP1 **(C)**, and TIMP2 **(D)** of relative band density in each treatment group normalized to stain-free image are shown below the Western blot images. Significant differences were determined by Student’s t-test. The individual points represent the ratios of proteins (n = 3), the horizontal lines represent the means and the error bars represent SEM. ** P < 0.01 **(A,C)**, * P < 0.05 **(B)**, **** P < 0.0001 **(D)**.

Changes in transcript and protein expressions of MMP2, MMP9, TIMP1 and TIMP2 were assessed following 48-h of THC exposure. The THC-stimulated cells showed a twofold reduction in *MMP2* (*P* < 0.0001) and *MMP9* (*P* < 0.01) transcripts concomitant with a 2.5- and 4.5-fold increase in *TIMP1* (*P* < 0.01) and *TIMP2* (*P* < 0.0001), respectively, when compared to unstimulated cells (Fig. 2). Similarly, we demonstrate a marked reduction in MMP2 and MMP9 (*P* < 0.01, *P* < 0.05, respectively; Fig. 3A,B) protein expression along with significantly increased protein expression of the inhibitors TIMP1 and TIMP2 (*P* < 0.01, P < 0.0001, respectively; Fig. 3C,D).

### Mitochondrial fission and fusion transcripts are significantly altered in response to THC

**Figure 4:**
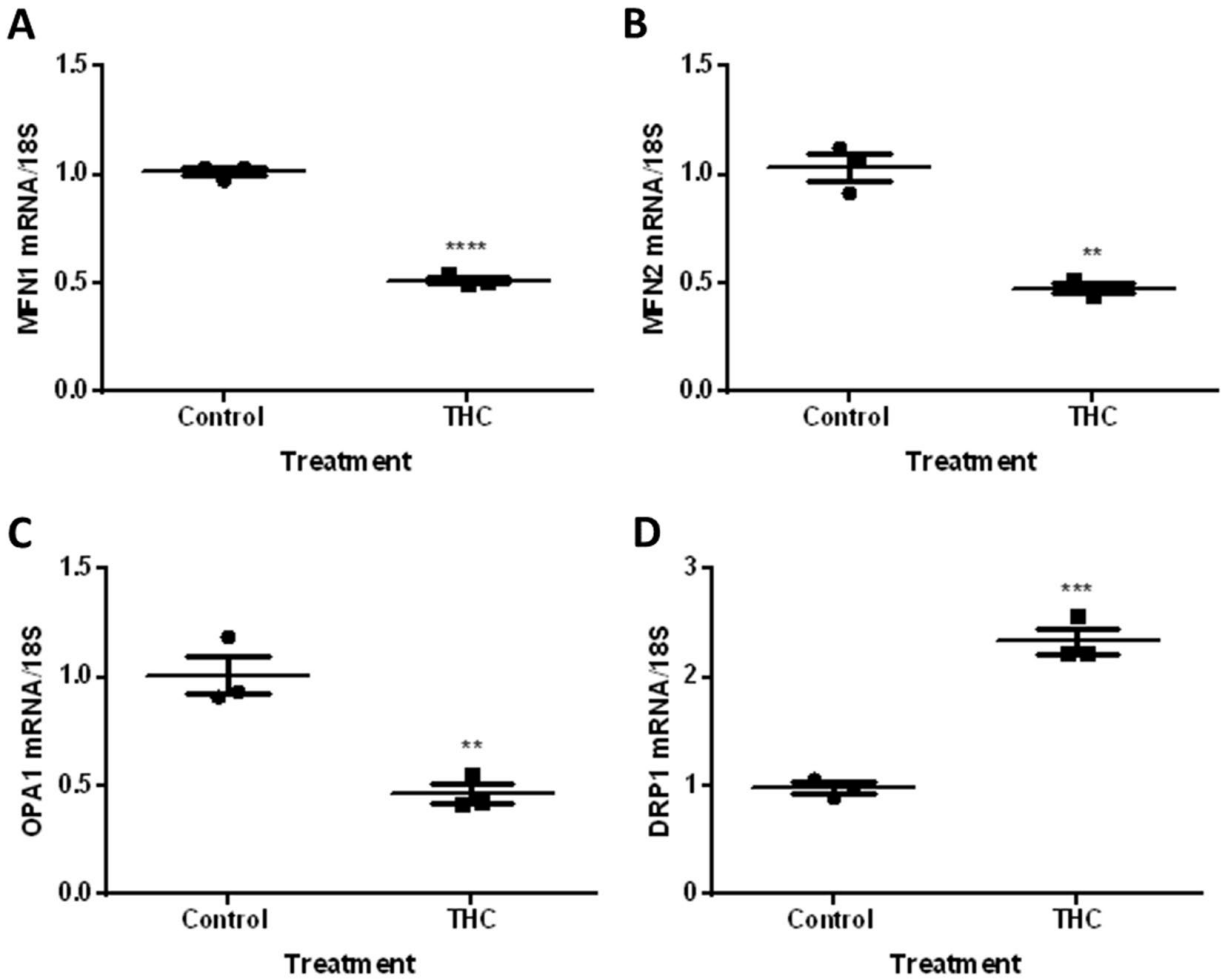
Mitochondrial fusion and fission transcripts are altered in response to 20 µM THC in HTR8/SVneo cells. Summary histograms are shown of relative *MFN1* **(A)**, *MFN2* **(B)** *OPA1* **(C)** and *DRP1* **(D)** transcript expression in each treatment group following 48 h of THC treatment which were normalized to 18S, then compared to the gene in the vehicle control group. Significant differences were determined by Student’s t-test. Individual data points are presented, the mean represented by the horizontal lines ± SEM (n = 3). **** *P* < 0.0001 **(A)**, ** *P* < 0.01 **(B,C)**, *** *P* < 0.001 **(D)**.

To investigate the effects of THC on altering markers that govern mitochondrial dynamics, we treated HTR8/SVneo cells for 48 h with 20 µM THC and assessed the transcript expression of key fission and fusion markers. In response to THC treatment, we demonstrate an approximately 50% reduction in *MFN1*, *MFN2*, and *OPA1* transcript expression, while *DRP1* transcript expression increased over twofold, relative to untreated control cells (Fig. 4, *P* < 0.0001, *P* < 0.01, *P* < 0.01, *P* < 0.001, respectively).

### Involvement of cannabinoid receptors in anti-invasive actions of THC

**Figure 5:**
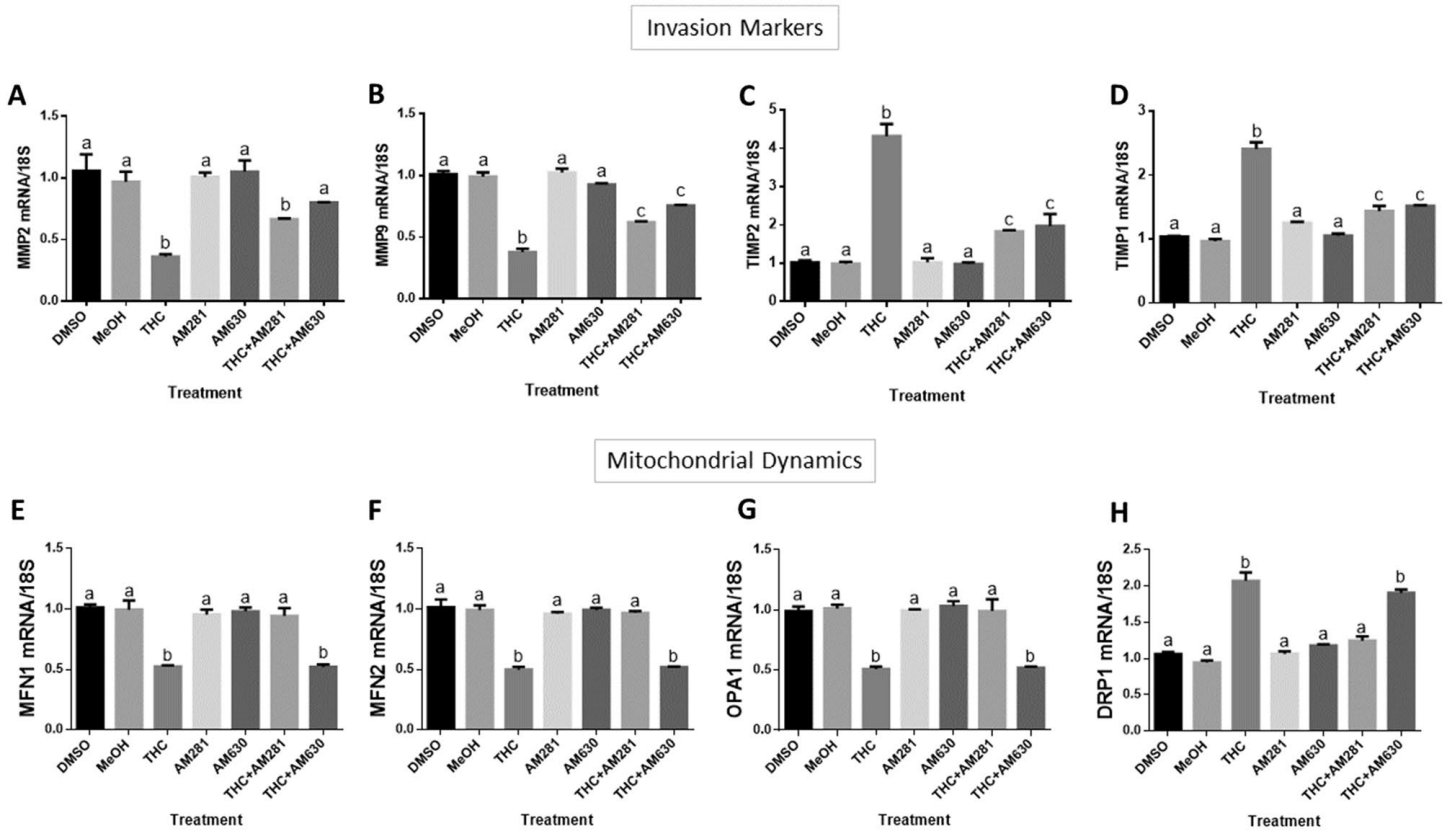
CBR-THC binding reduces invasive potential and disturbs mitochondrial dynamics in HTR8/SVneo cells. HTR8/SVneo cells were treated for 48 h as described in “Methods”, with DMSO and methanol (MeOH) used as vehicle controls. Summary histograms of relative *MMP2* **(A)**, *MMP9* **(B)**, *TIMP2* **(C)**, *TIMP1* **(D)** *MFN1* **(E)**, *MFN2* **(F)**, *OPA1* **(G)** and *DRP1* **(H)** transcript expression in each treatment group normalized to 18S, then compared to the gene in the vehicle controls. Significant differences were determined by a one-way ANOVA, followed by a Bonferroni post hoc test. Data are presented as means ± SEM (n = 3). Different letters denote significant differences compared to vehicle controls (**A–H**, *P* < 0.0001). THC: 20 µM; AM281: 1 µM; AM630: 1 µM.

We evaluated whether the canonical cannabinoid receptors are involved in THC-mediated reduction in HTR8/SVneo cell invasion using CB1 (AM281, 1 µM) and CB2 (AM630, 1 µM) antagonists. The reductions in *MMP2* transcripts that were observed with THC treatment were completely blocked by CB2 antagonism (*P* < 0.0001) while remaining unchanged in response to antagonizing CB1 (Fig. 5A). THC-induced suppression of *MMP9* transcripts was partially blocked following both CB1 and CB2 antagonism (Fig. 5B, P < 0.0001). At the same concentration of both CB1 and CB2 antagonists, the THC-induced transcription of *TIMP1* and *TIMP2* were partially blocked (Fig. 5C,D, P < 0.0001).

### Effects of CB1 and CB2 receptor antagonism on THC-mediated mitochondrial perturbation

HTR8/SVneo cells were treated with CB1 and CB2 antagonists to determine which receptors mediated the transcriptional changes in mitochondrial fission and fusion markers. THC-stimulated reductions in *MFN1*, *MFN2* and *OPA1* and the THC-stimulated increase in *DRP1* was completely blocked by CB1 antagonism (*P* < 0.0001). CB2 antagonism had no effect on these transcriptional markers (Fig. 5E–H).

### THC increases transcriptional markers of stress responses, ROS production and 4-HNE adducts in HTR8/SVneo cells without changing the intracellular defenses

**Figure 6:**
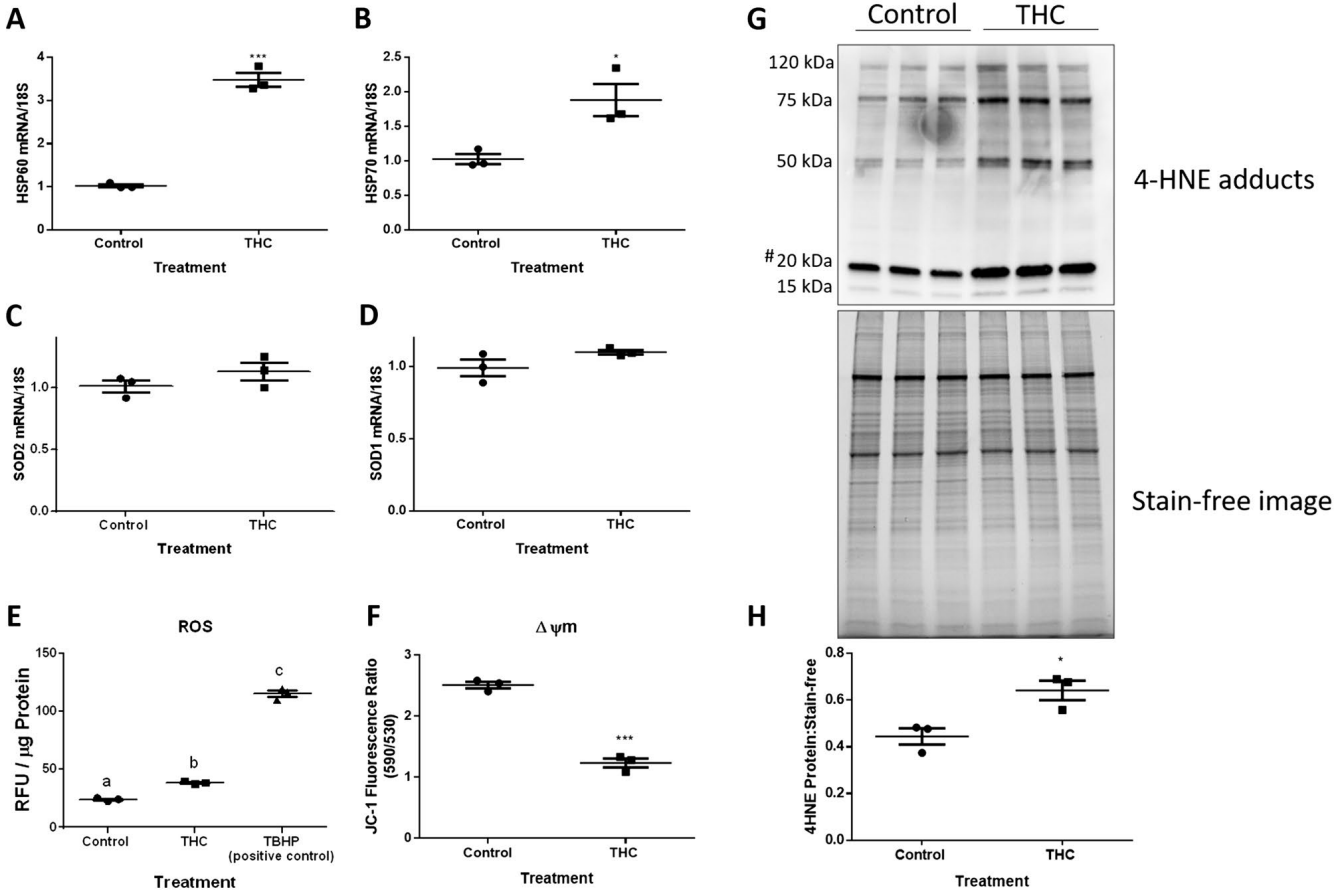
THC increases transcriptional markers of stress responses, ROS production and lipid peroxidation in HTR8/SVneo cells without changing the expression of superoxide dismutases. Summary histograms are shown of relative *HSP60* **(A)**, *HSP70* **(B)**, SOD2 **(C)**, and SOD1 **(D)** transcript expression in each treatment group following 48 h exposure to 20 µM THC which were normalized to 18S, then compared to the gene in the vehicle control group. **(E)** DCFDA assays were performed to determine intracellular ROS levels following THC treatment. Tert-Butyl Hydrogen Peroxide (TBHP) solution (100 µM) was used as the positive control. Results were normalized to the protein content of cell lysates via the BCA assay, which enables the standardization of ROS production as a function of cell number (protein level). **(F)** THC decreased JC-1 fluorescence ratio. **(G)** A western blot showing four major protein bands with molecular weights of approximately 120, 75, 50, 20 and 15 kDa showed immunoreactivity for 4-HNE modifications in control and treated trophoblast cells from whole cell lysates (20 µg). **(H)** Quantification of the density of the four major protein bands in the 4-HNE blot for each treatment group are shown as a ratio normalized to stain-free image. The hashtag (#) indicates a ~ 20 kDa band of proteins which show greater sensitivity to oxidative damage. The individual points represent measurements (n = 3), the horizontal lines represent the means and the error bars represent SEM. Significant differences were determined by Student’s t-test. *** *P* < 0.001 **(A,E)**, * *P* < 0.05 **(B,H)**, *P* = ns **(C,D)**, *P* < 0.001 **(F)**. *RFU* relative fluorescence units.

We further investigated whether oxidative stress is induced in trophoblast cells following THC treatment. THC exposure stimulated ROS production, as detected by DCF fluorescence (Fig. 6E). Treatment of HTR8/SVneo cells with 20 µM THC for 48 h significantly increased DCF fluorescence by 60%, compared with that of unstimulated control cells (*P* < 0.001).

Since THC increased ROS production in HTR8/SVneo cells, we investigated the effects of THC on intracellular stress responses and free radical defenses. THC treatment resulted in significant increases in heat shock protein (*HSP*) *60* and *70* (Fig. 6A, P < 0.001; 6B, *P* < 0.05) along with the detection of increased 4-HNE protein adduct levels (Fig. 6G,H, P < 0.05). While increased markers of cellular stress and oxidative damage were observed, there was no evidence of an increase in the level of mRNA encoding the antioxidant enzymes manganese superoxide dismutase and copper zinc superoxide dismutase (*SOD2* and *SOD1*) (Fig. 6C,D).

### OXPHOS proteins are decreased following THC exposure

**Figure 7:**
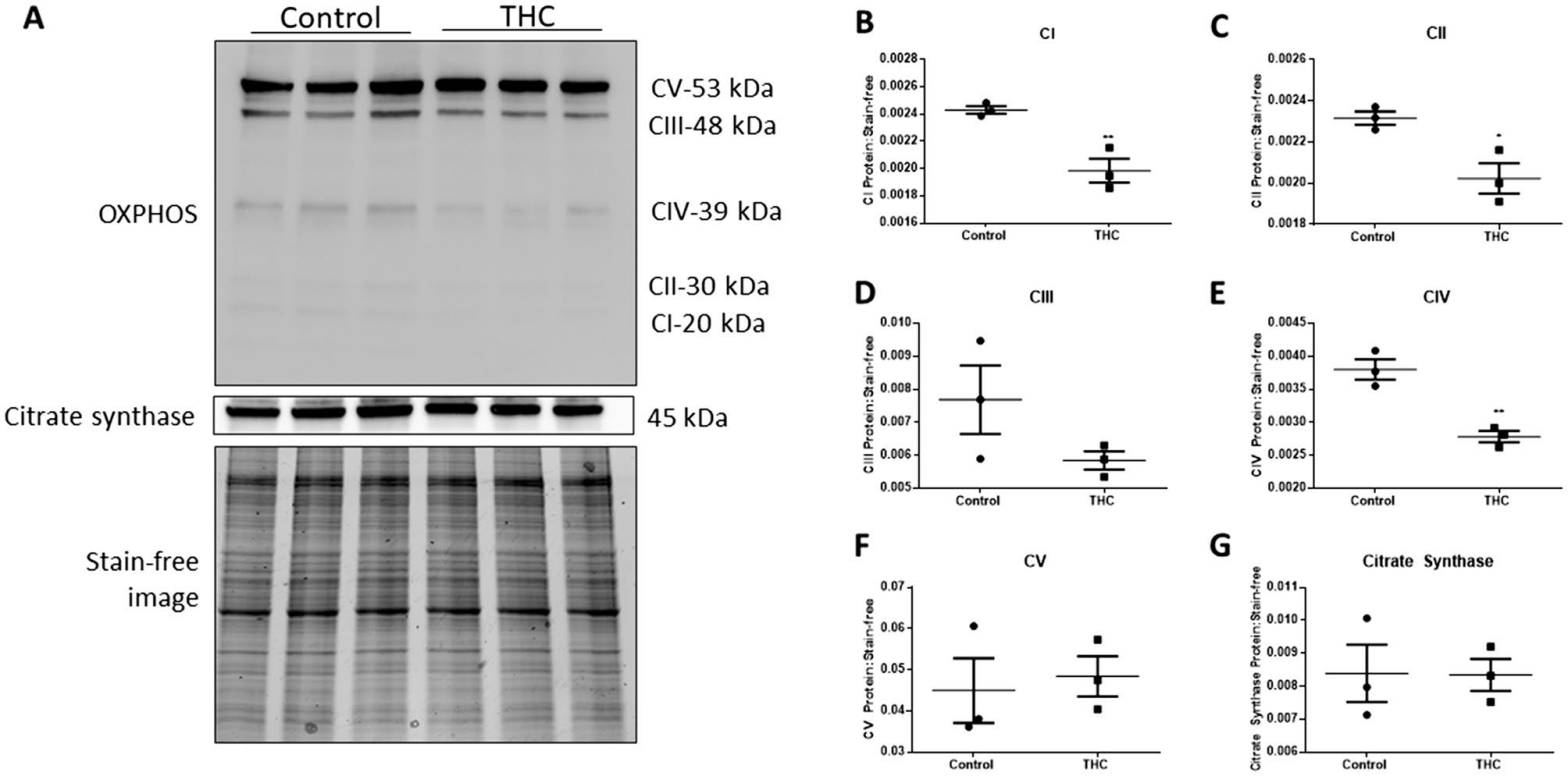
THC reduces levels of mitochondrial complex proteins without altering citrate synthase protein expression. **(A)** Western blot images (20 µg) of mitochondrial OXPHOS respiratory complex protein levels, citrate synthase and stain-free image in control and 20 µM THC-treated conditions after 48 h. A cocktail antibody comprising the following subunits of respiratory complex proteins was used: NADH dehydrogenase (ubiquinone) 1 beta subcomplex 8 (NDUFB8; complex I), succinate dehydrogenase complex, subunit B (SDHB; complex II), ubiquinol-cytochrome c reductase core protein II (UQCR2; complex III), cytochrome c oxidase subunit 2 (COXII; complex IV) and ATP synthase 5A (ATP 5A, Complex V). Quantification of the levels of each of the above-mentioned subunits and CS expression are shown, respectively **(B–G)** The individual points represent the ratios of proteins normalized to stain-free image (n = 3), the horizontal lines represent the means and the error bars represent SEM. Significant differences were determined by Student’s t-test. **P < 0.01 **(B,E)**; *P < 0.05 **(C)**; *P* = ns **(D,F,G)**.

Given our observed indicators of mitochondrial dysfunction, we next investigated whether THC decreased the expression of the proteins which comprise the electron transport chain. THC reduced the levels of mitochondrial OXPHOS protein subunits of the respiratory chain following 48 h of exposure (Fig. 7). Specifically, electron transport chain (ETC) complex protein subunits NADH dehydrogenase (ubiquinone) 1 beta sub complex 8 (NDUFB8; complex I, *P* < 0.01), succinate dehydrogenase complex, subunit B (SDHB; complex II, *P* < 0.05), and cytochrome c oxidase subunit 2 (COXII; complex IV, *P* < 0.01) were significantly reduced. Ubiquinol-cytochrome c reductase core protein II (UQCR2; complex III) and ATP synthase 5A (ATP 5A, Complex V) were not significantly changed in response to 20 µM THC relative to untreated controls. The citrate synthase protein level, a proxy for mitochondrial mass^46^, was not significantly altered relative to untreated controls following 48 h of THC treatment (Fig. 7A,G).

### THC causes mitochondrial membrane depolarization in HTR8/SVneo cells

Treatment with 20 µM THC for 48 h significantly decreased the JC-1 red/green fluorescence intensity ratio by 50.8% in HTR8/SVneo cells compared to untreated controls (Fig. 6F, P < 0.001).

### THC reduces electron transport chain function in HTR8/SVneo cells

**Figure 8:**
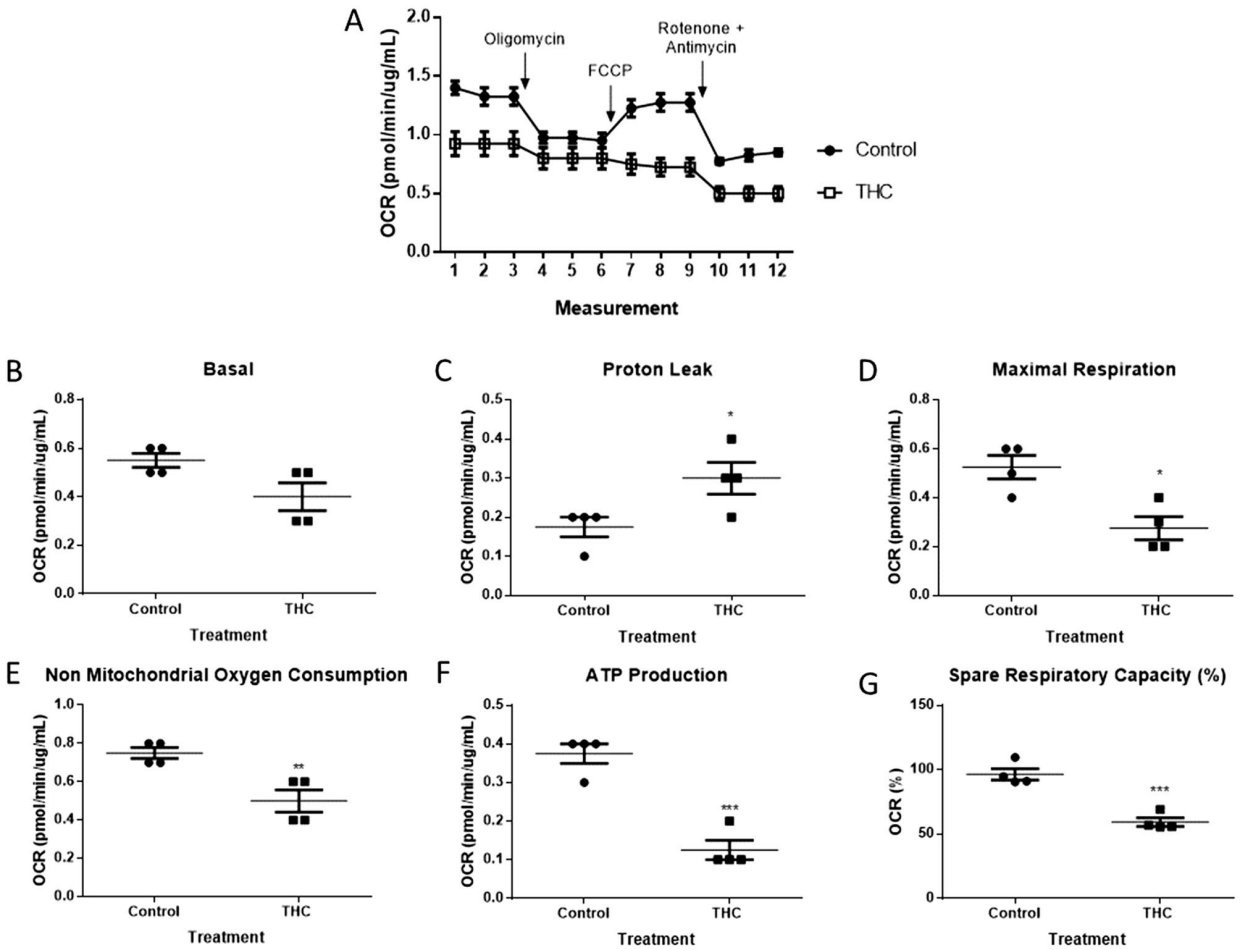
Mitochondrial respiration indices are impaired in HTR8/SVneo cells upon 48-h exposure to 20 µM THC. **(A)** Representative OCR tracing. **(B–G)** Mitochondrial parameters as indicated. The detection of OCR was performed with 4 biological replicates per experiment, for each treatment condition, and repeated 3 more times (see Supplemental Fig. 2 for experiments 2–4). Individual data **(B–G)**, group mean and SEM **(A–G)** are shown. Significance was assessed by Student’s t-test (*P < 0.05, **P < 0.01, ***P < 0.001).

Since mitochondrial function is directly linked to mitochondrial membrane polarization state^47^, we investigated the changes in mitochondrial aerobic metabolism that occurred in HTR8/SVneo cells in response to THC. A Cell Mito Stress assay kit was used to detect the OCR. HTR8/SVneo cells were treated with 20 µM THC for 48 h before exposure to 1 µM oligomycin, 2 µM FCCP and 0.5 µM rotenone and antimycin. As demonstrated in Fig. 8A, THC reduced OCR in HTR8/SVneo cells. THC did not significantly change basal respiration (Fig. 8B). THC significantly reduced maximal respiration (Fig. 8D, 47.6%, *P* < 0.05), non-mitochondrial oxygen consumption (Fig. 8E, 33.3%, *P* < 0.01), ATP production (Fig. 8F, 66.7%, *P* < 0.001) and spare respiratory capacity (Fig. 8G, 38.5%, *P* < 0.001), while significantly increasing proton leak (Fig. 8C, 71.4%, *P* < 0.05) when compared to the untreated cells.

## Discussion

THC impacts a number of physiological processes^2,48^. Here, we provide insight into the effects of THC on impairing human trophoblast invasion, a critical step in the placentation process. Several studies employing THC in the µM range (up to 30 µM), representative of moderate to heavy cannabis consumption^49^, have shown that THC inhibits cytotrophoblasts proliferation and transcription of genes encoding proteins involved in processes such as, apoptosis and ion exchange^25^, trophoblast migration and invasion^50^, as well as angiogenesis in the placentae of cannabis users^51^. Importantly, we have previously shown that THC impairs cytotrophoblast fusion into syncytia, hormonal secretion, and mitochondrial function, i.e. reduced ATP production and increased ROS production^26^.

Our present work demonstrates that THC reduced the ability of HTR8/SVneo cells to proliferate and invade due to changes in the transcript levels of genes that play important roles in this process. In addition, THC-induced ROS production was accompanied by disruption of mitochondrial dynamics in HTR8/SVneo cells. Mitochondrial morphology is determined by a dynamic equilibrium between organelle fusion and fission^52^. Perturbation of mitochondrial dynamics, as demonstrated by alterations in the transcriptional markers used in this study, has been linked to altered trophoblast function^26,30,41^. Importantly, increases in mitochondrial fission have been associated with poor gestational outcomes^31^. Our previous study using human placental BeWo cells^26^ demonstrated that THC treatment increased markers of mitochondrial fission and negatively impacted the secretion of hormones critical for fetal growth.

The reduction in the invasive capacity of HTR8/SVneo trophoblast cells can be attributed, in part, to the observed reduction in the transcription and protein expression of MMPs. In support of this, THC and endogenous cannabinoids have been shown to interact with cancer cells to negatively impact growth and proliferation^53–59^. Like tumour cells, invasiveness is a feature of trophoblasts. However, unlike tumour invasion, trophoblast invasion is a strictly controlled physiological event^27,60^. Excessive trophoblast invasion may result in placenta accreta^61,62^, while insufficient invasion of the trophoblast may result in PE^63^. Trophoblast invasion into the decidual spiral arteries is crucial for normal placental development and pregnancy success. Impaired spiral artery remodelling, which has been associated with attenuated trophoblast invasion^28,64^ can lead to a failure to accommodate high capacity blood flow; an outcome that has been associated with a host of pregnancy-related pathologies^27^.

The observed attenuation of invasion can be a consequence of reduced MMP activity. While we did not quantify metalloproteinase activity, we do demonstrate that THC-mediated a significant reduction in transcription of the genes encoding *MMP2* and *MMP9* as well as their protein expression levels. The gelatinase MMPs (MMP2 and MMP9), are secreted as proenzymes that are subsequently activated by proteolytic processing^63^ and permit digestion of the extracellular matrix (ECM) and surrounding tissue^27^ in order to facilitate trophoblastic decidual invasion^28^. In breast cancer and glioma cells, using in vitro and in vivo methods, treatment with THC resulted in reductions in MMP2 expression and activity^58,59^. Furthermore, a direct link between cannabinoid receptor activation and MMP expression has been identified by Adhikary et al. (2012). Using selective CB receptor agonists GP1a or O-1996, these researchers identified that CB2 receptor signaling reduced migration of bone marrow-derived dendritic cells in both in vitro and in vivo (murine) models, primarily by inhibiting expression of MMP9^65^. In addition, Blázquez et al. (2008) demonstrated reduced *MMP2* transcript levels in glioma cells in response to 24 h THC exposure, which was completely reversed by CB2 antagonism, but not by CB1 antagonism^59^; a finding that is reflected in our observations. Moreover, Ramer and Hinz (2008) used human cervical cancer cells (HeLa) to assess invasiveness following treatment with an endogenous cannabinoid (anandamide) analog R( +)-methanandamide (MA) and THC with or without CB1 and CB2 receptor antagonists. The authors report that both MA and THC reduce invasiveness of the HeLa cells concomitant with increased expression of TIMP1 transcript and protein, all of which were suppressed upon pre-treatment with CB1 and CB2 receptor antagonism^66^. Similarly, we also observed increased gene and protein expression of TIMP1 and TIMP2, proteins which are known to attenuate MMP2 and MMP9 proteolytic activity^63,66^. Taken together, our results suggest that the THC-mediated reduction in invasive activity of trophoblasts may be due to reduced MMP function.

Following 48-h exposure to THC, we sought to determine mitochondrial respiration measurements in HTR8/SVneo cells. An increase in proton leak and a decrease in mitochondrial respiration, as we have demonstrated in HTR8/SVneo cells, are indicative of mitochondrial dysfunction^67^. Mitochondrial oxygen consumption was increased by uncoupling the mitochondria via the addition of FCCP. This resulted in an increase in the maximal respiratory response. Following the delivery of rotenone and antimycin, inhibiting the mitochondrial ETC, we observed the loss of the enhanced OCR, thus confirming the portion of the profile that was linked with mitochondria-specific respiration. While the bulk of oxygen is consumed by mitochondria, enzymatic reactions and oxygenases outside of mitochondria will also contribute to the total cellular oxygen consumption^68^. We conclude that overall cellular metabolism is decreased as both mitochondrial and non-mitochondrial oxygen consumption are reduced in response to THC. Further, FCCP-induced maximal respiration allows the determination of spare respiratory capacity (%), defined as the difference between maximum (uncoupled) OCR and basal OCR^67^, which is reduced in the HTR8/SVneo cells exposed to THC. Spare respiratory capacity can be used as a surrogate readout for the ability of the mitochondria to increase oxygen consumption in response to an increased demand for ATP. Supporting our evidence, a recent study by Lojpur et al., using the BeWo choriocarcinoma cell line, demonstrated that 24-h treatment with 15 µM THC negatively impacted mitochondrial respiration as assessed via OCR, in part due to reduced expression of proteins associated with the mitochondrial respiratory chain complexes^22^. Our observations also suggest that THC treatment reduces expression of subunit proteins associated with complexes I, II and III. Other studies have also demonstrated that THC disrupted mitochondrial function in other cell types, although this was observed with concentrations of THC that were greater than that used in our work (up to 120 µM and approximately 50 µM, respectively)^69,70^.

Monitoring mitochondrial membrane potential in parallel with respiration, both of which were reduced following 48-h THC exposure, allowed for a more powerful and informative measure of mitochondrial dysfunction^68^. Decreased mitochondrial membrane potential (decreased JC-1 fluorescence ratio) is linked to increased mitochondrial recycling^71^. Additionally, increased ROS production has also been associated greater mitochondrial fission and formation of smaller, more punctate mitochondria^72^. These smaller structures, independent of the reticulum-like structure that is associated with healthy cellular function, are thought to have altered cristae structure and be characterized by less efficient ETC activity^73^. In support of this, we observed increased expression of transcripts indicative increased mitochondrial fragmentation (*DRP1)* and decreased expression of genes encoding proteins involved in promoting mitochondrial fusion. Importantly, while we demonstrate increased mitochondrial fission, the overall mitochondrial mass may not be changing, as indicated by the lack of change in the steady state levels of CS in our system. It is possible that a pool of more fragmented mitochondria are contributing to less efficient ATP production, as indicated by reduced expression of OXPHOS enzymes, and reduced ATP production and OCR. Furthermore, this pool of mitochondria may be an important source of oxidative stress and contribute to increased lipid peroxidation in trophoblast cells^74^.

Oxidative stress, described as ROS production which exceeds the ability of antioxidant defenses to scavenge them, is an important contributing factor in the pathophysiology of complicated pregnancies^75,76^. Thus, oxidative stress can result from increased ROS production and/or defects in antioxidant defense systems^76^. Oxidative damage occurs from the interaction of free radicals with DNA and intracellular macromolecules such as proteins and membrane lipids, including lipid peroxidation, subsequently leading to cellular dysfunction^77^. Although a physiological balance between ROS and antioxidant defense systems is maintained in uncomplicated pregnancies, an imbalance may increase oxidative stress^76^. Indeed, in response to THC exposure, HTR8/SVneo cells demonstrate a significant increase in intracellular stress responses (*HSP60* and *HSP70* transcripts), concomitant with an increase in oxidative stress. However, antioxidant defenses (*SOD1* and *SOD2*) remained unchanged relative to control. The overall lack of change in the expression of SODs may contribute to the significant increase in ROS in our study, thus precipitating significant oxidative stress. Indeed, Akhigbe and Id (2020) treated male rabbits with codeine (4 mg/kg and 10 mg/kg, for 6 weeks) and assessed testicular antioxidant enzyme activities and demonstrated marked increases in oxidative stress without the concomitant increase in SOD activity^77^. Downstream of the induction of oxidative stress is the initiation of peroxidation and subsequent lipid dysfunction^78^. Physiological levels of ROS are normally managed by the activation of SOD enzymes as well as the ensuing damage elicited by lipid peroxidation^78^. However, when protective systems are overwhelmed by oxidative stress, this may lead to various pathological changes^77–79^. Indeed, following THC treatment, HTR8/SVneo cells demonstrate marked increases in ROS production and the expression of the lipid aldehyde, 4-HNE, which is a marker of lipid peroxidation. We demonstrate that exposure of these trophoblasts to 20 µM THC for 48 h overwhelmed the inherent defense systems, as SOD enzyme expressions were not changed.

Considering that metabolic impairment has been linked to the pathophysiology of several placental disorders^30,31^, we speculate that THC-induced decreases in mitochondrial respiratory chain functioning and increased mitochondrial fission mediated by CB1 could be an important mechanism of action of THC. Interestingly, inhibition of CB2 receptor did not significantly impact the effects of THC on the markers of mitochondrial fragmentation. We have made similar observations in syncytialized BeWo cells treated with THC^26^. Drawing from the “double-hit” hypothesis^80^ in the context of trophoblast physiology, THC users may have compromised mitochondrial function such that they will less effectively handle the impact of a subsequent stressor on the mitochondrial reticulum (such as poor maternal nutritional status)^80^.

## Conclusion

Our results suggest a potential cascade of molecular changes that occur in the placenta upon exposure to THC. We have demonstrated that THC impairs trophoblast invasion by reducing MMP expression and increasing TIMP expression, mediated in part by the canonical CB receptors. We further demonstrate that THC adversely affects the function of human trophoblasts by altering mitochondria-dependent pathways leading to increased organellar fission, reduced OCR, and aberrant ROS production. Furthermore, recently published data from our group demonstrates that THC reduces the molecular signals for BeWo syncytialization and the expression of growth factors important for fetal growth^41^. Taken together, these observations suggest that this cannabinoid alters the process of placentation through its influence, in part, on trophoblasts function. Recent work from Natale et al. (2020) also demonstrate that THC can alter angiogenesis in placenta and reduce birth weight in the offspring of rat dams injected with THC during pregnancy^23^. Importantly, our work also suggests that mitochondria, via the CB1 receptor, may have a role in mediating the effects of THC. Our work contributes to the understanding of the cellular mechanisms underpinning the adverse pregnancy outcomes associated with cannabis use during gestation.

## Methods

### Cell culture

All cell culture experiments were carried out under McMaster University Biohazard Utilization Protocol BUP023. HTR8/SVneo cells (a kind gift from Dr. Peeyush K. Lala, Professor in the Department of Oncology at Western University, London, ON, Canada) were grown and maintained in RPMI-1640 medium (Lonza, 12-115F, Walkersville, MD, USA) supplemented with 5% FBS, 1% penicillin/streptomycin, and 1% L-glutamine, maintained in a humidified atmosphere of 5% CO_2_ at 37 °C.

### Cell viability assays

Cell viability assays were conducted as previously described by our group^41^. Briefly, HTR8/SVneo cells were seeded into a 96-well plate at a density of 10,000 cells/well. Control wells containing media without cells were allocated to determine background absorbance. Cells were treated with THC (Sigma, Cat. No. T4764) or vehicle (methanol; MeOH) for 48 h.

For the MTS Assay, the cells were treated with 20 µL of CellTiter 96 AQueous Non-Radioactive Cell Proliferation Assay (Promega Corp., Cat. No. G5421) for 2 h at 37 °C in a humidified, 5% CO_2_ atmosphere. The absorbance was recorded at 490 nm using a 96-well plate reader (Miltiskan Spectrum spectrophotometer; Thermo Scientific, Canada). The results are normalized to the untreated cells and plotted as a percent of control.

As a measure of plasma membrane integrity, lactate dehydrogenase (LDH) release into culture supernatants was detected spectrophotometrically at 490 nm and 680 nm, using the Pierce LDH Cytotoxicity Assay Kit (Cat. No. 88953), according to the manufacturer’s recommended protocol. The results are presented as fold increase in the absorbance measured normalized to untreated cells.

### DCFDA (2′,7′-dichlorofluorescin diacetate) assay

HTR8/SVneo cells were seeded in a black, clear bottom 96-well microplate at a cell density of 30,000 cells/well. The cells were treated with 100 µL of media supplemented with 20 µM THC or vehicle for 48 h. The cells were assayed using the Abcam DCFDA Cellular ROS Detection Assay Kit (Abcam Cat. No. ab113851) according to the manufacturers recommended protocol. Cells were analyzed on a fluorescent plate reader (BioTek Synergy 4) read in endpoint mode at excitation and emission wavelengths of 485 and 535 nm, respectively. Data were standardized as a percent of control after background (blank wells with media only) subtraction, followed by normalization to total protein content (BCA)^81^.

### Transwell invasion assay

Transwell invasion assays were carried out as per manufacturers instructions (Corning). Briefly, Matrigel invasion assays were carried out at 37 °C for 48 h using 24-well transwell inserts with 8.0 μm pores (Corning Cat. No. 353097) coated with 0.3 mg/mL of GFR Matrigel (Corning, Cat. No. 354230). In the apical chambers, 50,000 cells were seeded on top of the Matrigel and supplemented with 500 µL of serum-free RPMI-1640 containing vehicle or THC (10 μM or 20 μM), while 750 µL media with chemoattractant (5% FBS) supplemented with vehicle or THC was placed into the basal chambers. Following 48 h, non-invading cells and Matrigel were gently removed from the apical side of the membrane using a cotton swab moistened with cold phosphate buffered saline (PBS). Cells that migrated and invaded through the membrane were fixed in ice-cold 100% MeOH for 5 min at − 20 °C and incubated with 4′, 6-diamidino-2-phenylindole (DAPI, 1.5 µg/mL, diluted in PBS with Tween and 0.1% bovine serum albumin (BSA)) for 5 min at room temperature and visualized at 200× magnification with a Nikon Eclipse Ti-E (Nikon Instruments Inc., USA). Images were captured using NIS Elements AR v5.11.01. Five non-overlapping fields of view were captured per sample. The number of cells that traversed the Matrigel and membrane were counted by two individuals who were blinded to the treatment groups using ImageJ software (National Institutes of Health, Bethesda, MD, USA). The mean number of cells were determined, and the percent invasion was expressed as the mean number of invaded cells exposed to the drug relative to untreated cells.

### RNA extraction and RT-PCR

Following 48 h of treatment with 20 µM THC, HTR8/SVneo cells grown on 12-well plates were lysed with 500 µL of ice-cold TRIzol reagent (Thermo Fisher Scientific, Canada). Total RNA isolation and quantification of gene expression (RT-PCR) was performed as previously described by our research team^82^. All genes and their respective primer sequences are listed in Table 1. Fold change transcript expression was quantified using the double delta Ct (ΔΔCt) analysis, normalized to housekeeping gene, *18S*, then expressed as the relative fold change to the vehicle control sample expression.

**Table 1:** Primer sequences of human genes analyzed via RT-PCR.

Gene	Forward	Reverse	GenBank
18S	CACGCCACAAGATCCCA	AAGTGACGCAGCCCTCTATG	NR_003286.2
DRP1	AAACTTCGGAGCTATGCGGT	AGGTTCGCCCAAAAGTCTCA	NM_012062.5
HSP60	GAAGGCATGAAGTTTGATCG	TTCAAGAGCAGGTACAATGG	NM_002156.5
HSP70	GGAGTTCAAGAGAAAACACAAG	AAGTCGATGCCCTCAAAC	NM_005345.6
MFN1	TTGGAGCGGAGACTTAGCAT	GCCTTCTTAGCCAGCACAAAG	NM_033540.3
MFN2	CACAAGGTGAGTGAGCGTCT	ACCAGGAAGCTGGTACAACG	NM_014874.4
MMP2	TCTCCTGACATTGACCTTGGC	CAAGGTGCTGGCTGAGTAGATC	NM_004530.5
MMP9	CCGGCATTCAGGGAGACGCC	TTGAACCACGACGCCCTTGC	NM_004994.2
OPA1	GCTCTGCATACATCTGAAGAACA	AGAGGCTGGACAAAAGACGTT	NM_130831.3
SOD1	AAAGATGGTGTGGCCGATGT	CAAGCCAAACGACTTCCAGC	NM_000454.5
SOD2	GCTCCGGTTTTGGGGTATCT	GATCTGCGCGTTGATGTGAG	NM_001024465.3
TIMP1	GGGCTTCACCAAGACCTACA	TGCAGGGGATGGATAAACAG	NM_003254.2
TIMP2	GAAGAGCCTGAACCACAGGT	GGGGGAGGAGATGTAGCA	NM_003255.4

### BCA assay

Protein concentration was determined by using the bicinchoninic assay (BCA; ThermoFisher, Canada) with BSA (0–2000 µg/mL) as a concentration standard. Total protein concentration was measured with a 96-well plate reader (Miltiskan Spectrum spectrophotometer; Thermo Scientific, Canada) set at A562 nm.

### SDS-PAGE and western blotting

HTR8/SVneo cells treated with 20 µM THC for 48 h were lysed in radioimmunoprecipitation assay (RIPA) lysis buffer containing protease inhibitor cocktails (Roche Diagnostics, Cat. No. 04693159001). Samples were loaded for gel electrophoresis at 20 µg/sample and all gels (12%) were imaged using the stain-free application on the ChemiDoc (Bio-Rad) imager immediately after the protein separation and prior to western blotting. Protein gels were blotted using the Trans-Blot Turbo transfer apparatus PVDF Midi transfer packs (Bio-Rad). Antibody conditions are listed in Table 2. Immunoreactive bands were visualized using Clarity Max Western ECL Substrate (Bio-Rad) and visualized using ChemiDoc Imaging System (BioRad, V2.3.0.07). The intensities of the bands were quantified using Image Lab (BioRad, V6.0.1). The Image Lab values generated for the proteins of interest were normalized to total protein on the same stain-free membrane. Full blots available in the Supplementary Information files, Figure S3(A–I).

**Table 2:** Antibodies used for western blot.

Antibody	Manufacturer	Catalogue number	Host organism	Blocking medium (in TBST)	Dilution	Antibody dilutant (in TBST)	RRID
4-HNE	Abcam	ab48506	Mouse	5% BSA	1:2000	3% BSA	AB_867452
CS	A kind gift from Dr. B.H Robinson, Hospital for Sick Children, Toronto ON	N/A	Rabbit	5% BSA	1:10,000	3% BSA	N/A
MMP-2	Abcam	ab51125	Rabbit	5% milk	1:1000	5% milk	AB_881239
MMP-9	GeneTex	GTX61537	Rabbit	5% milk	1:1000	5% milk	AB_10619391
OXPHOS	MitoSciences/Abcam	MS601-360	Mouse	5% BSA	1:1000	5% BSA	AB_1619331
TIMP-1	GeneTex	GTX112096	Rabbit	5% milk	1:1000	5% milk	AB_11174643
TIMP-2	GeneTex	GTX21828	Mouse	5% milk	1:1000	5% milk	AB_372322

### CB1 and CB2 antagonism

To address the role of the canonical cannabinoid receptors, CB1 and CB2, in mediating the effects of THC, selective antagonists AM281 and AM630^83^, respectively, were used at a final concentration of 1 µM each^84^. These receptor antagonists were used at a concentration of 1 µM, which is within the range of concentrations that have been reported to inhibit cellular responses to activation of the canonical receptors^84,85^.

HTR8/SVneo cells were pre-incubated with the antagonists for 30 minutes^84^, followed by treatment with 20 µM THC for 48 h.

### Mitochondrial respiration assay

Cellular energetics were measured using an XFe24 Extracellular Flux Analyzer (Agilent, Santa Clara, CA, USA) as previously described by our group^26^. The mitochondrial oxygen consumption rate (OCR) was measured at 37 °C in an XFe24 Extracellular Flux Analyzer (Agilent, Santa Clara, CA, USA). Crucial for this assay, cell density was optimized. HTR8/SVneo cells were plated at a density of 60,000 cells/well in 250 µL culture media, in 24-well microtiter plates and allowed to adhere overnight. The day before running the XF Assay, the Seahorse XF Sensor Cartridge was hydrated by adding 1 mL of sterilized double distilled water to each well of the XF Utility Plate. The hydrated cartridge was kept in a non-CO_2_ 37 °C incubator overnight to remove CO_2_ that would otherwise interfere with measurements that are pH sensitive. The day of the assay, the water was replaced with Agilent XF calibrant (pH 7.4) for a minimum of 1 h in a non-CO_2_ 37 °C incubator. HTR8/SVneo cells were exposed to 20 µM THC for 48 h. On the day of the XF Assay, culture media was aspirated and replaced with XF base medium (Seahorse Bioscience, Cat. No. 102365-100) supplemented with 100 mM sodium pyruvate (ThermoFisher Scientific, Cat. No. 11360-070), 200 mM l-glutamine (ThermoFisher Scientific, Cat. No. 25030081), 5 mL of 45% glucose solution (Millipore-Sigma, Cat. No. G8769), warmed to 37 °C (adjusted to pH 7.4). OCR was detected under basal conditions followed by the sequential injections of 1 μM oligomycin (ATP-synthase inhibitor), 2 μM carbonyl cyanide-4-(trifluoromethoxy)phenylhydrazone (FCCP; mitochondrial respiration uncoupler), and 0.5 μM rotenone combined with antimycin (electron transport blockers). The OCR value measured after oligomycin treatment indicates the amount of oxygen consumption linked to ATP production, and that after FCCP injection represents the maximal mitochondrial respiratory capacity of the cells. The final injection of rotenone and antimycin inhibits the flux of electrons through complex I and III, respectively, and thus no oxygen is further consumed at complex V. The OCR reading after this treatment is primarily non-mitochondrial. OCR measurements were obtained using the Seahorse XFe24 Analyzer and the OCR values were normalized to the amount of protein content from each well. The detection of OCR was performed with four biological replicates per experiment, for each treatment condition, and repeated three more times.

### Mitochondrial membrane potential

THC-stimulated changes in the mitochondrial membrane potential (ΔΨm) were assessed using the fluorescent reagent tetraethylbenzimidazolylcarbocyanine iodide (JC-1) with the JC-1-Mitochondrial Membrane Potential Assay kit (Abcam, Cat. No. ab113850) following the manufacturer’s protocol, and as previously described by our group^26^. HTR8/SVneo cells were seeded at a density of 50,000 cells/well and allowed to adhere overnight in a black, clear-bottom 96 well plate. Cells were treated with 20 µM THC for 48 h. Following treatment, cells were washed once with 1× dilution buffer and then incubated with 20 µM JC-1 dye in 1× dilution buffer for 10 min at 37 °C, protected from light. JC-1 dye was then removed, cells were washed once with 1× dilution buffer, 100 µL of fresh 1× dilution buffer was added to each well. The red fluorescence in excitation (535 nm)/emission (590 nm) and green fluorescence excitation/emission (475 nm/530 nm) was measured using a Spark multimode microplate reader (Tecan Group Ltd.). Background fluorescence was subtracted from the fluorescence of treated cells, then the ratio of red (polarized) fluorescence divided by that of green (depolarized) fluorescence was obtained.

### Statistical analyses

All experiments were performed in biological triplicates or quadruplets. Comparisons between vehicle control and THC-treated cells were performed using Student’s t-test. One-way analysis of variance (ANOVA) and Bonferroni post hoc tests were used to compare data sets with more than two groups. Data are reported as means ± SEM. Differences were considered significant at *P* < 0.05. The experimental parameters were analyzed using XFe Wave software V2.6.1 and Excel (for OCR measurements) and GraphPad Prism software V6.0.

## Supplementary Information

Supplementary Figures.

## Data Availability

All data generated or analysed during this study are included in this published article (and its supplementary information files).
