# Supplementary material for: Delta-9-tetrahydrocannabinol inhibits invasion of HTR8/SVneo human extravillous trophoblast cells and negatively impacts mitochondrial function
Source: Sci Rep. 2021 Feb 17;11:4029. doi: 10.1038/s41598-021-83563-9 (PMC7889882; doi:10.1038/s41598-021-83563-9)
Supplement: Supplementary file 1 — Supplementary Figures. [file 41598_2021_83563_MOESM1_ESM.docx]

**Delta-9-tetrahydrocannabinol inhibits invasion of HTR8/SVneo human extravillous trophoblast cells and negatively impacts mitochondrial function**

**Supplementary Material**

O’Llenecia S. Walker^1^, Harmeet Gurm^1^, Reeti Sharma^2^, Navkiran Verma^2^, Linda L May^2^, Sandeep Raha^1, *^


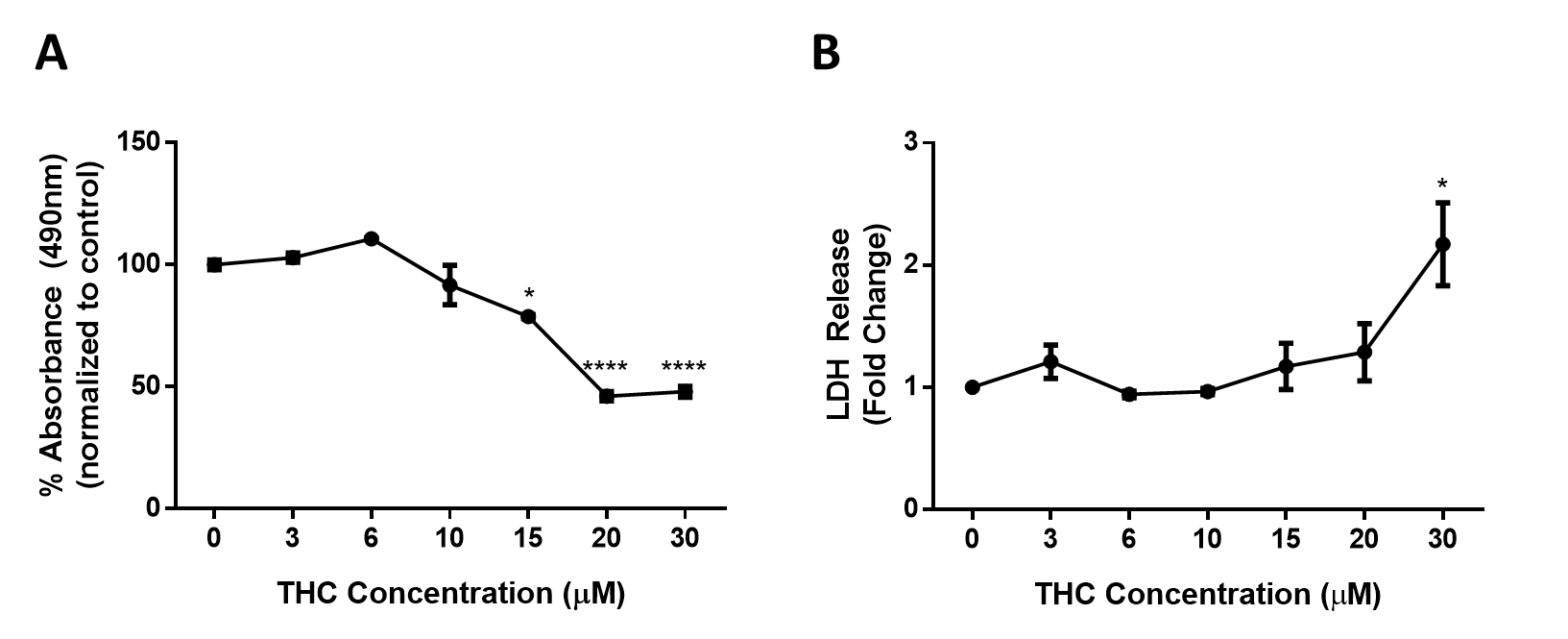


**Figure S1. THC negatively impacts HTR8/SVneo cells viability and plasma membrane integrity.** HTR8/SVneo cells were treated with THC as indicated for 48 hours and subjected to MTS **(A)** and LDH **(B)** assays. Each data point represents the mean ± SEM of 3 biological replicates measured at 490nm (MTS) or 490nm and 680nm (LDH). Significant differences were determined by a one-way ANOVA followed by a Bonferroni post hoc test. **P* < 0.05; *****P* < 0.0001, relative to control.


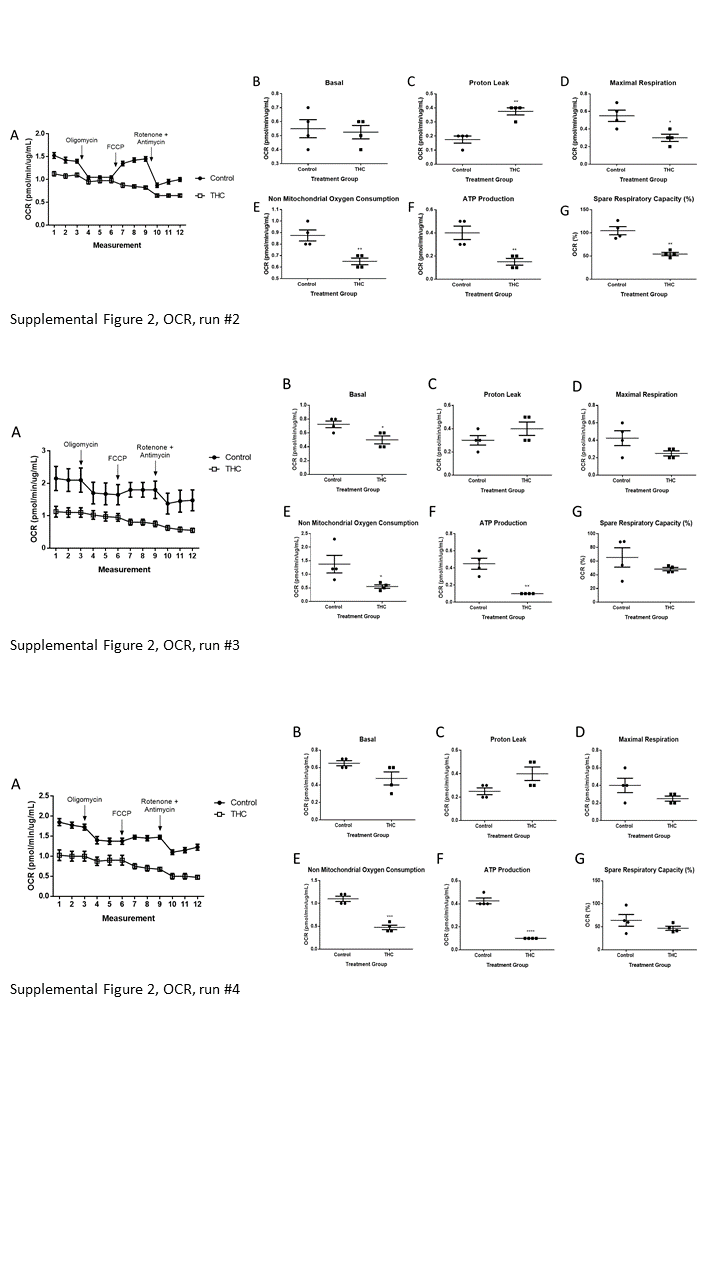


**Figure S2. Mitochondrial respiration indices are impaired in HTR8/SVneo cells upon 48-hour exposure to 20 µM THC (experiments 2-4).** **(A)** Representative mitochondrial profile. **(B-G)** Mitochondrial parameters as indicated. The detection of OCR was performed with 4 biological replicates per experiment, for each treatment condition, and repeated 3 more times. Individual data (B-G), group mean and SEM (A-G) are shown. Significance was assessed by Student’s t-test (**P* < 0.05, ***P* < 0.01, ****P* < 0.001).

**A**

**
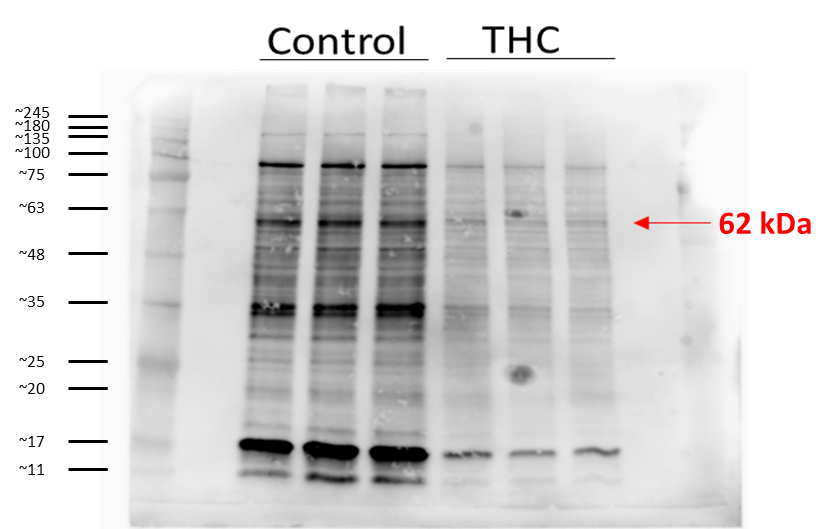
**

**B**

**
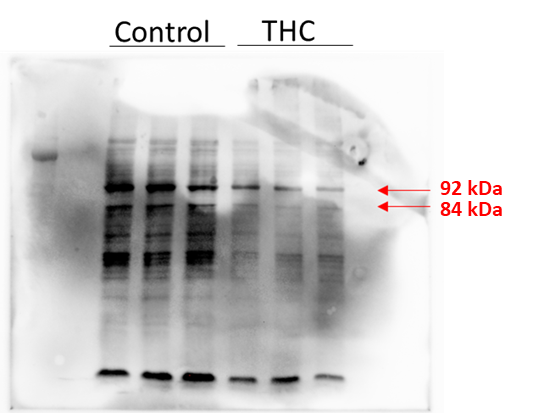
**

**C**

**
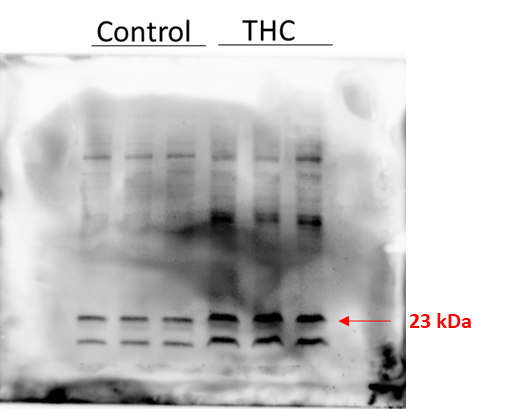
**

**D**

**
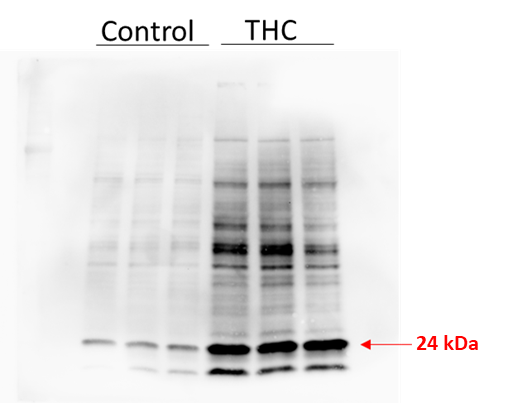
**

**E**

**
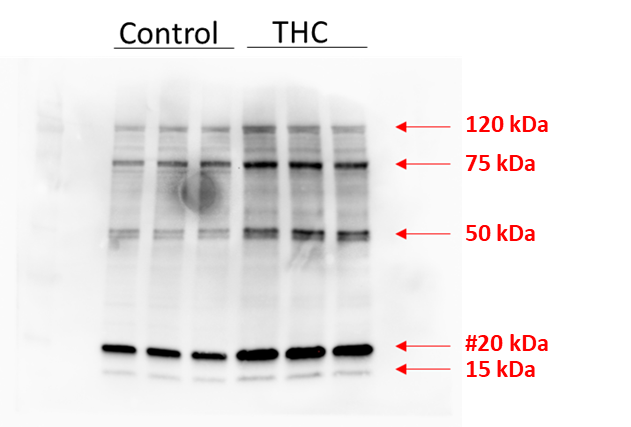
**

**F**


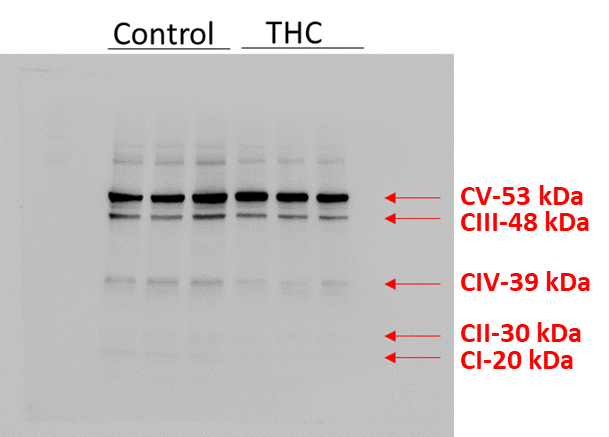


**F**


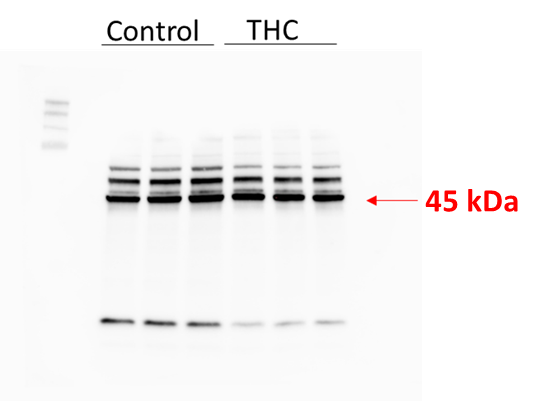


**F** **G**


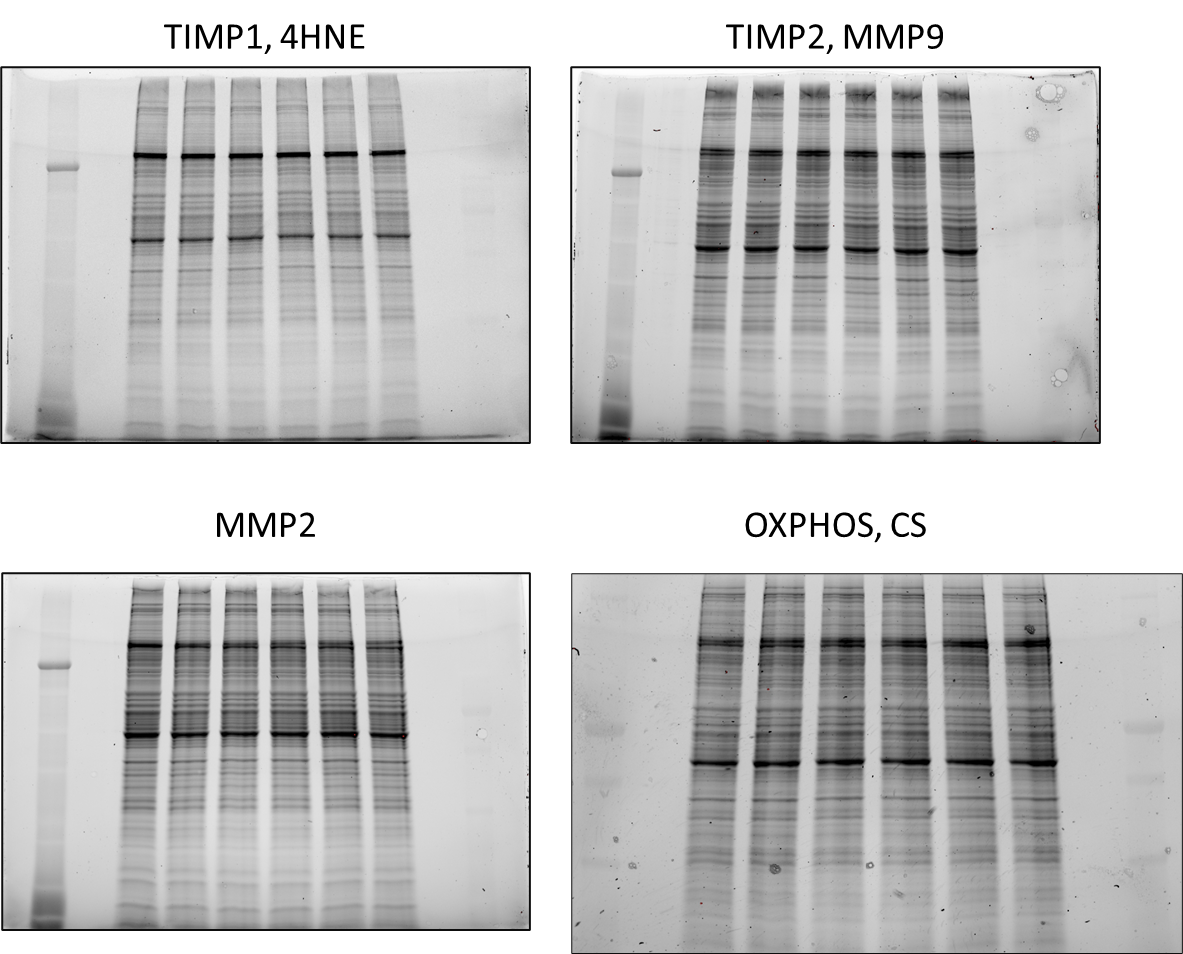


**H I**


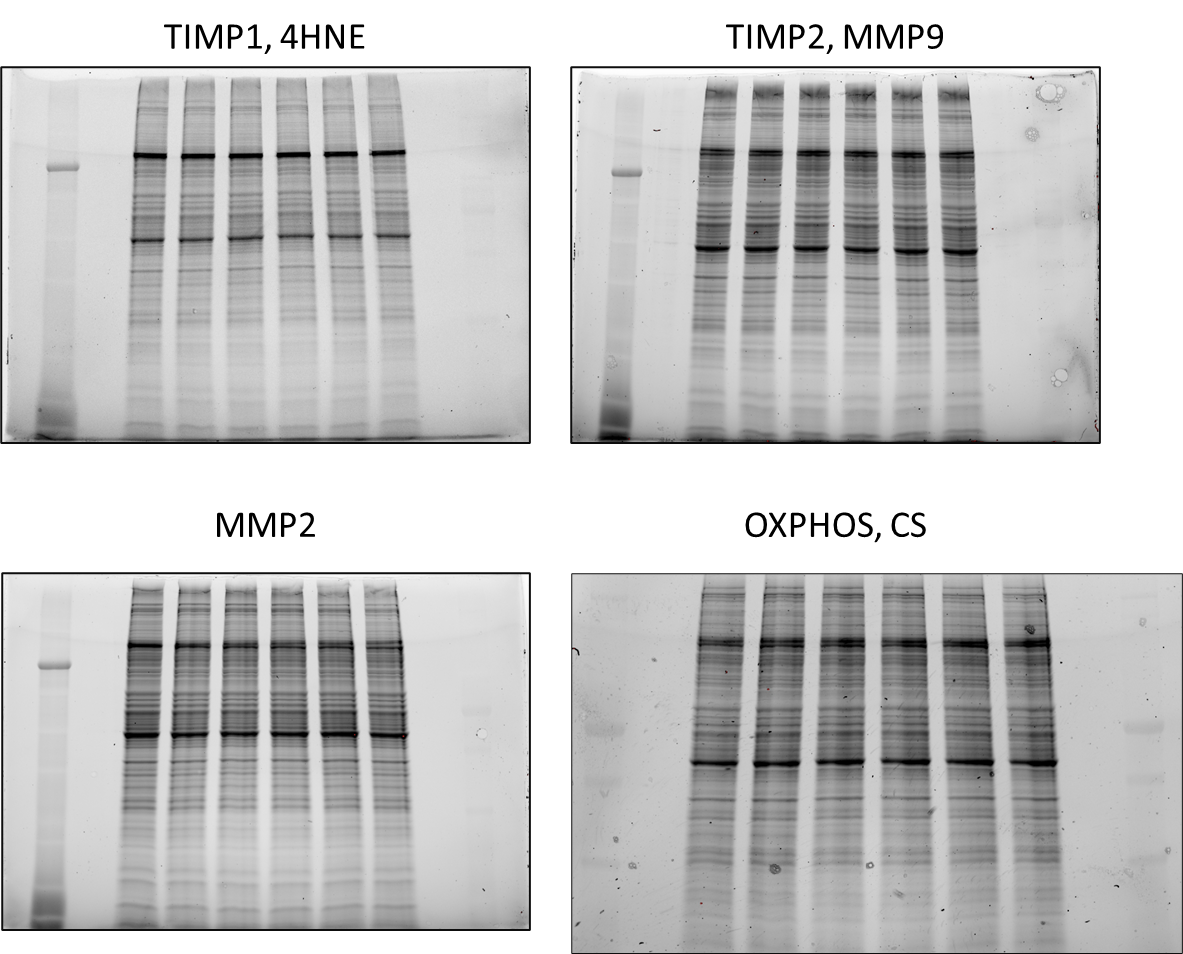


**Figure S3. Representation of full Western blot images and stain-free gel images.** Uncropped Western blots images representative of the figures utilized in the primary manuscript are provided here. **(A)** MMP2; **(B)** MMP9; **(C)** TIMP1; **(D)** TIMP2; **(E)** 4-HNE; **(F)** Oxidative Phosphorylation enzymes; **(E)** Citrate Synthase (CS). Total protein visualized using Stain-Free gels (BioRad Laboratories) which were used for the Western blots in panels A-E. **(F)** Representation of the SDS PAGE used for the TIMP1 and 4-HNE Western blots. **(G)** Representation of the SDS PAGE used for the TIMP2 and MMP9 Western blots. **(H)** Representation of the SDS PAGE used for the MMP2 Western blot. **(I)** Representation of the SDS PAGE used for the Oxidative Phosphorylation enzymes and CS Western blots.
